# Female sex mitigates motor and behavioural phenotypes in TDP-43^Q331K^ knock-in mice

**DOI:** 10.1038/s41598-020-76070-w

**Published:** 2020-11-05

**Authors:** Jodie Watkins, Anshua Ghosh, Amy F. A. Keerie, James J. P. Alix, Richard J. Mead, Jemeen Sreedharan

**Affiliations:** 1 Department of Neuroscience, School of Medicine, Sheffield Institute for Translational Neuroscience, University of Sheffield, Sheffield, S10 2HQ UK; 2 Maurice Wohl Clinical Neuroscience Institute, Institute of Psychiatry, Psychology and Neuroscience, King’s College London, 5 Cutcombe Road, London, SE5 9RX UK

**Keywords:** Amyotrophic lateral sclerosis, Dementia, Neuroscience, Diseases, Medical research, Neurology

## Abstract

Amyotrophic lateral sclerosis (ALS) and frontotemporal dementia (FTD) are overlapping neurodegenerative disorders. ALS is more commonly seen in men than women and the same may be the case for FTD. Preclinical models demonstrating sex-specific vulnerability may help to understand female resistance to ALS-FTD and thereby identify routes to therapy. We previously characterised a TDP-43^Q331K^ knock-in mouse, which demonstrated behavioural phenotypes reminiscent of ALS-FTD in males. Here we present our behavioural observations of female TDP-43^Q331K^ mutants. Female TDP-43^Q331K^ knock-in mice displayed increased weight relative to wild-type and increased food intake at 20 months of age, much later than previously observed in male mutants. Spontaneous digging behaviour was initially normal and only declined in mutants in the second year of life. Gait analysis using Catwalk (https://www.noldus.com/catwalk-xt) found significant deficits in the second year of life, while nocturnal running behaviour was attenuated from ~ 250 days of life. These results indicate that while female TDP-43^Q331K^ knock-in mice do display progressive behavioural phenotypes, these are less severe than we previously noted in male mutants. Further studies of male and female TDP-43^Q331K^ knock-in mice may help to unravel the mechanisms underlying sex-specific vulnerability in ALS-FTD.

## Introduction

Amyotrophic lateral sclerosis (ALS) and frontotemporal dementia (FTD) are neurodegenerative disorders with shared clinical, neuropathological and genetic features, indicating that they lie on a continuum^1–3^. While pure ALS is characterised by motor impairment, including weakness, FTD is defined by cognitive and behavioural dysfunction. A greater understanding of the mechanisms driving neurodegeneration is needed if we are to develop therapies for these devastating conditions.

Interestingly, ALS has long been recognised to disproportionately affect males. Both the incidence and prevalence of ALS are greater in men than in women and men also have a younger age of onset^4–6^. There are also phenotypic differences between the sexes, with a predominance of limb onset ALS in men and bulbar onset in women^4,6^. Epidemiological studies of FTD are fewer in number and smaller in scale, making its sex-specific effects more difficult to determine. However, a handful of studies suggest that FTD is more prevalent in males^7,8^, and can cause more brain hypometabolism in men when the severity of cognitive symptoms is controlled for^9^. These effects can, however, vary between geographical populations^10–12^. Bulbar onset ALS together with cognitive impairment specifically shows an overwhelmingly male predominance^13^. Taken together, these observations indicate an important but undefined role for biological sex in dictating the specific phenotype as well as the risk of developing diseases of the ALS-FTD spectrum.

Almost all patients with ALS, and 50% of all FTD cases, are characterised neuropathologically by nuclear clearing and cytoplasmic mislocalisation of the 43-kDa transactive response DNA-binding protein (TDP-43)^14,15^. Over 50 disease-associated mutations have been found in *TARDBP*, the gene encoding TDP-43, which account for ~ 5% of fALS, < 1% of sALS, and rare familial cases of FTD^16–19^. The majority of these mutations are clustered in the glycine-rich C-terminal domain^20^ and, interestingly, some show greater penetrance in men than women^21^. Although the roles of TDP-43 in disease development are elusive, the use of preclinical models that recapitulate aspects of human disease have helped to define key underlying mechanisms. While transgenic approaches may be confounded by artefacts caused by spatiotemporal patterns and levels of expression that do not faithfully recapitulate the normal biology of a protein, recently developed knock-in models that manipulate endogenous alleles are more likely to accurately represent the human condition^22^. We recently characterised a TDP-43^Q331K^ knock-in mouse model of ALS-FTD harbouring only a human equivalent missense mutation in the endogenous murine *Tardbp* gene^23^. In our analysis of breeding ratios, we found that while female TDP-43^Q331K^ knock-in mutants were present at Mendelian ratios, male mutants were under-represented. This suggested that males were more susceptible to deleterious effects of the TDP-43^Q331K^ mutation^23^. We subsequently focussed our attention on male mice, finding that male mutants displayed FTD-like deficits^23^, including executive dysfunction, weight gain due to hyperphagia^24^, and reduced digging behaviour suggestive of apathy^25^. However, whether similar phenotypes occurred in female mutants and whether these are attenuated, as they are in women compared to men, was not determined.

Here, we present results from a longitudinal study to investigate behaviour in female homozygous mutant (TDP-43^Q331K/Q331K^) knock-in mice and wild-type littermates. Given the increased incidence and severity of ALS-FTD in men^4–7^, and the increased penetrance of *TARDBP* mutations in males^21^, we tested the hypothesis that female sex attenuates disease caused by mutant TDP-43^Q331K^.

## Results

### Weight gain and age-dependent increase in food intake in female mutant mice

**Figure 1:**
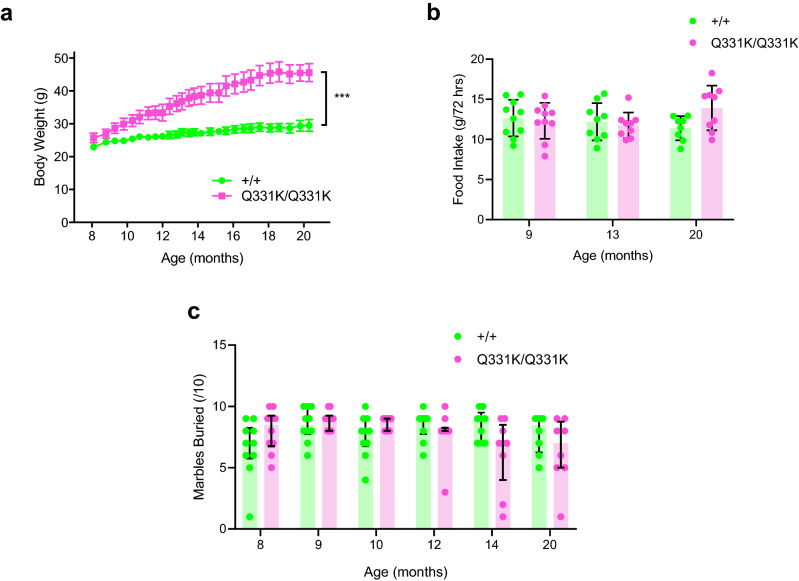
Female TDP-43^Q331K/Q331K^ mice show marked weight gain over time, and age-dependent trends towards increased food intake and reduced marble burying. (**a**) Body weight from 8 to 21 months of age (n = 6–10 wild-type, 8–10 mutants) is significantly increased in mutants (overall *P* = 0.0005; mixed-effect analysis). (**b)** Food intake at 9, 13 and 20 months of age (n = 10 per genotype (9 months); 9 wild-type, 8 mutants (13 months); 8 wild-type, 9 mutants (20 months)) (overall *P* = 0.3260; two-way ANOVA). (**c)** Marble burying from 8 to 20 months of age (n = 10 per genotype (8, 9, 10 and 12 months); 9 per genotype (14 months); 8 per genotype (20 months)) shows no significant difference between groups (*P* = 0.6689; unpaired t-test). Error bars represent mean ± s.e.m, except in (**c)** where they are median ± interquartile range. ***P < 0.001.

We previously showed that male TDP-43^Q331K/Q331K^ mice displayed increased weight gain from 8 months of age and were also hyperphagic compared to wild-type mice^23^. We therefore weighed female wild-type and TDP-43^Q331K/Q331K^ mice but found that at 8 months of age there were no significant differences in weight (23.0 ± 1.7 g and 25.7 ± 3.8 g, respectively, *P* = 0.9692). However, TDP-43^Q331K/Q331K^ mice gradually gained more weight over time and were significantly heavier than wild-type mice from 13 months of age. By 20 months, wild-type mice weighed 29.5 ± 5.0 g and TDP-43^Q331K/Q331K^ mice weighed 45.6 ± 7.7 g (*P* < 0.01; mixed-effects analysis) (Fig. 1a). To determine if weight gain in the TDP-43^Q331K/Q331K^ mice could be due to hyperphagia, food intake was measured when mice were 9, 13 and 20 months old. The amount of food eaten in 72 h did not change with age and there was no significant difference between groups, although at 20 months there was a trend towards increased food intake (Fig. 1b). Wild-type and TDP-43^Q331K/Q331K^ mice ate 12.7 ± 2.3 g and 12.3 ± 2.2 g of food at 9 months of age (*P* = 0.9801), 12.2 ± 2.3 g and 11.8 ± 1.6 g of food at 13 months of age (*P* = 0.9633) and 11.4 g ± 1.5 g and 13.9 ± 2.8 g at 20 months (*P* = 0.0601 two-way ANOVA), respectively.

### Age-dependent deficits in marble burying behaviour in mutant females

A subset of male TDP-43^Q331K/Q331K^ mice were previously found to display reduced spontaneous digging as determined by the marble burying assay from as early as 5 months of age^23^. This may reflect apathy or reduced motivation, both of which are features of FTD^25^. We therefore examined marble burying behaviour in female wild-type and TDP-43^Q331K/Q331K^ mice from 8 to 20 months of age but found no significant differences between genotypes at any given age although there was a trend towards reduced digging from 14 months onwards (Fig. 1c). At 9 months of age, wild-type mice buried 8.5 ± 1.4 marbles and TDP-43^Q331K/Q331K^ mice buried 8.9 ± 0.7 marbles (*P* = 0.9966), whereas at 14 months wild-type mice buried 8.4 ± 1.2 marbles and TDP-43^Q331K/Q331K^ mice buried 6.2 ± 2.9 marbles (*P* = 0.0495). When mice reached 20 months of age wild-type mice buried 7.75 ± 1.6 marbles and TDP-43^Q331K/Q331K^ mice buried 6.4 ± 2.7 marbles (*P* = 0.5513, mixed-effects analysis). These results indicate that innate exploratory digging behaviours are largely intact in female mutant mice, although they may be affected in older age.

### Gait deficits in aged mutant females

**Figure 2:**
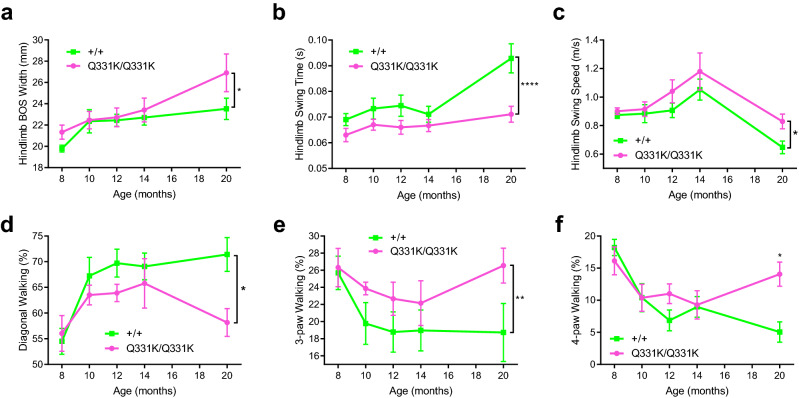
Catwalk gait analysis of female wild-type and TDP-43^Q331K/Q331K^ mice shows gait deficits in aged mutants. (**a)** Hindlimb base of support (BOS) (*P* < 0.05; two-way ANOVA). (**b)** Hindlimb swing time (*P* < 0.0001; two-way ANOVA). (**c)** Hindlimb swing speed (*P* < 0.05; two-way ANOVA). (**d)** Percentage of diagonal walking (*P* < 0.05; two-way ANOVA). (**e)** Percentage of 3-paw walking (*P* < 0.01; two-way ANOVA). (**f)** Percentage of 4-paw walking (*P* < 0.05 at 20 months on multiple comparisons; two-way ANOVA). All data is from 8 to 20 months of age (n = 10 per genotype (8 months); 10 wild-type, 9 mutants (10 and 12 months); 9 per genotype (14 months); 9 wild-type, 7 mutants (20 months)). Error bars represent mean ± s.e.m. *P < 0.05, **P < 0.01, ****P < 0.0001.

Our previous study showed that male TDP-43^Q331K/Q331K^ mice have reduced Rotarod performance from ~ 6 months of age, which was likely due to increased body weight rather than impaired motor coordination^23^. To test for motor coordination in female mice, we carried out detailed gait analysis using the Catwalk gait analysis system (Noldus, https://www.noldus.com/catwalk-xt). TDP-43^Q331K/Q331K^ mice had normal hindlimb base of support (BOS, the distance between the hind paws during the step cycle) from 8 to 14 months of age, but significantly wider hindlimb BOS at 20 months of age, when compared to wild-type mice (26.9 ± 5.3 mm and 23.5 ± 2.6 mm, respectively; overall *P* < 0.05, two-way ANOVA) (Fig. 2a). While this could be due to impaired motor coordination it could also be explained by the increased body weight.

TDP-43^Q331K/Q331K^ mice also showed decreased hindlimb swing time (time between successive paw placements) and increased swing speed (average speed of paw travelling during the swing phase). The hindlimb swing time and swing speed of wild-type mice at 20 months were 0.09 ± 0.01 s and 0.65 ± 0.12 m/s, respectively, and were 0.07 ± 0.01 s and 0.83 ± 0.16 m/s for TDP-43^Q331K/Q331K^ mice. This suggests that TDP-43^Q331K/Q331K^ mice walk faster than wild-type mice (Fig. 2b, c). However, the overall duration of the runs and hindlimb stride length showed no significant difference between the two groups at 20 months of age (data not shown). Together with swing speed and time, these results suggest that TDP-43^Q331K/Q331K^ mice take faster steps than wild-type mice.

Under normal conditions mice walk in a diagonal stepping pattern with only two diagonally opposed paws on the surface at any one time^26^. An increase in the percentage of time spent on 3 or 4 paws indicates instability whilst walking. Female wild-type mice spent the majority of their time on diagonal paws, and relatively little time on 3 and 4 paws from 10 to 20 months of age (Fig. 2d–f). In contrast, TDP-43^Q331K/Q331K^ mice spent significantly more time on 3 and 4 paws (Fig. 2 d–f). This difference was most pronounced at 20 months of age, by which time wild-type mice were spending 71.4 ± 8.8% of time on diagonal paws and 5.04 ± 4.1% of time on 4 paws, whereas mutants spent 58.1 ± 8.1% of time on diagonal paws and 14.1 ± 5.6% of time on 4 paws. These changes indicate that TDP-43^Q331K/Q331K^ mice stand for longer durations of the step cycle and may develop a less steady gait with age.

### Reduced running in mutant mice

**Figure 3:**
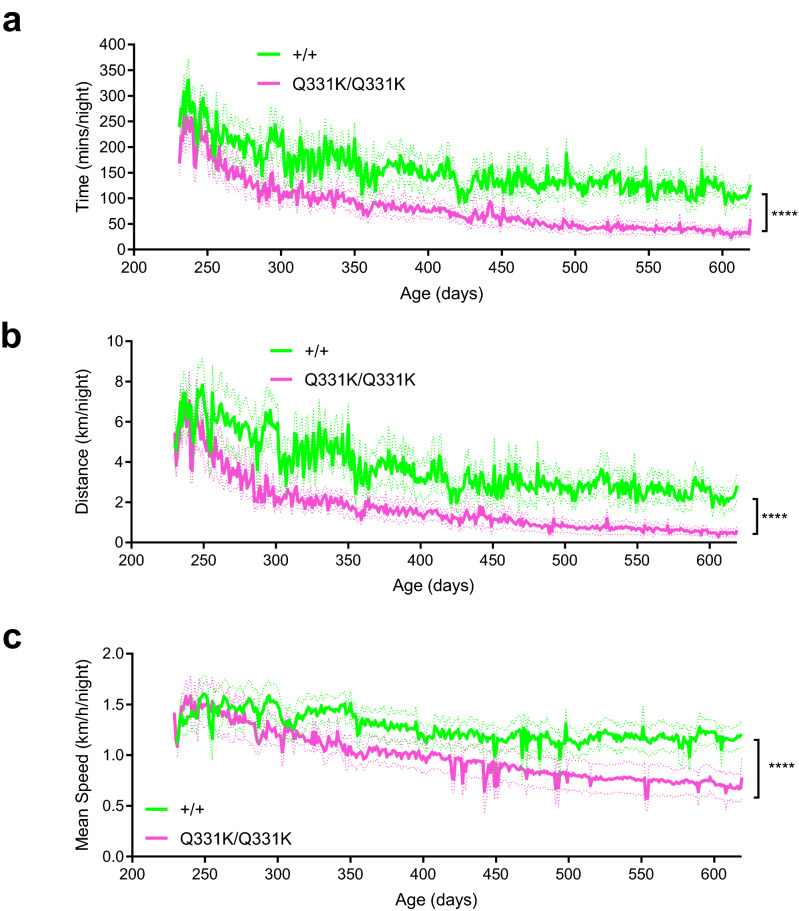
Running wheel analysis of female wild-type and TDP-43^Q331K/Q331K^ mice shows reduced running in mutants. (**a**) Time run per 24 h (*P* < 0.0001; two-way ANOVA). (**b)** Distance run per 24 h (*P* < 0.0001; two-way ANOVA). **c** Mean speed run per 24 h (*P* < 0.0001; two-way ANOVA). All data are from 230 to 618 days of age (n = 8–10 wild-type, 7–10 mutants). Error bars represent mean ± s.e.m. ****P < 0.0001.

To gain further insight into the motor performance of female TDP-43^Q331K/Q331K^ mice we measured voluntary wheel running, an assay that can be performed without disruption to the normal murine diurnal rhythm in a minimally stressful environment^27^. Behaviour was monitored daily in mice that had unlimited access to a running wheel, which was linked to a sensor to measure time spent running, total distance run, and speed. As they aged, both wild-type and TDP-43^Q331K/Q331K^ mice showed a progressive decline in total time and distance run per night, but this deterioration was more marked in mutants (Fig. 3a, b). Wild-type mice had a decrease from 286 ± 102 to 109 ± 56 min of running per night and mutants had a decrease from 252 ± 107 to 33 ± 30 min of running per night between 8 and 20 months of age (overall *P* < 0.0001, two-way ANOVA). Wild-type mice retained their average speed from 250 to 600 days of age, but mutants declined over time and became significantly slower. By 20 months of age wild-type mice were running 2.2 ± 1.81 km per night at 1.12 ± 0.35 km/h, whereas TDP-43^Q331K/Q331K^ mice were running 0.50 ± 0.61 km per night at 0.68 ± 0.38 km/h (Fig. 3c). This suggests that female TDP-43^Q331K/Q331K^ mice have reduced physical performance compared to wild-types.

### Preservation of functional motor units in mutant mice

**Figure 4:**
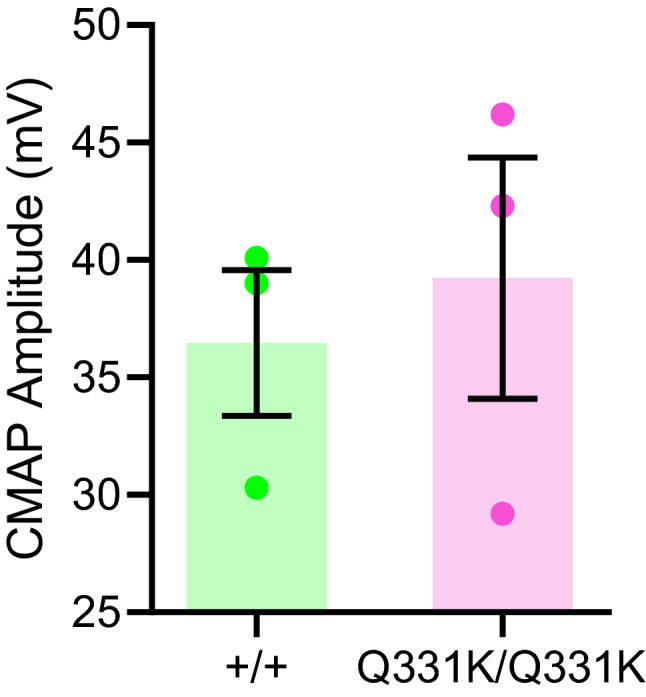
Compound muscle action potentials in 12-month-old mutant mice are no different to wild-type. n = 3 per genotype. *P* = 0.6689, unpaired t-test. Error bars represent mean ± s.e.m.

To determine if the motor deficits in female mutants were due to neuromuscular dysfunction, we looked for evidence of denervation by measuring compound muscle action potentials (CMAP) in the hindlimbs of 12-month-old mice. However, we found no significant differences in CMAP amplitudes between wild-type and mutant females (36.5 ± 5.4 mV and 39.2 ± 8.9 mV, respectively; n = 3 per genotype; *P* = 0.6689, unpaired t-test) (Fig. 4). This suggests that functional motor units are preserved in mutant mice and that the motor deficits they display are not due to neuromuscular denervation. Nonetheless, a more detailed analysis with a larger sample size and at more timepoints may be of value in determining if female mutants are vulnerable to denervation.

## Discussion

Epidemiological studies show that women are significantly less likely to develop ALS-FTD than men. Preclinical models of ALS-FTD that recapitulate this sex difference could help to understand the reasons for the protection conferred by female sex, which may in turn help towards developing therapies. In this study we found that female TDP-43^Q331K^ knock-in mice develop motor and behavioural deficits similar to those previously observed in male mutants, but that these phenotypes were less severe in females (Table 1). The clearest indicator of this was the striking weight gain seen in mutants, which occurred later in life and to a lesser extent in females than in males. Similarly, while food intake and marble burying behaviour were significantly increased and decreased respectively in male mutants, female mutants showed only trends towards such changes. The gradual increase in weight gain in both male and female TDP-43^Q331K/Q331K^ mice may be attributable to the role of TDP-43 in fat metabolism regulated by *Tbc1d1*^28,29^, which is most likely independent of oestrogen^30^. However, the hyperphagia observed in female mice at 20 months when oestrogen levels have dropped^31^ may directly result from release of the appetite stimulating gut hormone ghrelin, normally under tonic inhibition by oestradiol^32^. Thus, further studies of TDP-43^Q331K^ mice promise to help unravel how female sex protects against ALS-FTD.

**Table 1:** Summary of differences between male and female TDP-43^Q331K^ mice in age of onset of behavioural phenotypes, compared to wild-type.

Phenotype (compared to wild-type)	Age of onset (months)	
	Male^23^	Female
Weight gain	8	13
Increased food intake	9^a^	20^b^
Reduced marble burying	5	14^b^
Gait defects	6	20
Reduced running	n/a^c^	8
Muscle denervation	None	None

^a^Earliest age at which food intake was measured.

^b^Statistical trend.

^c^As male mice on the C57BL/6 background have low baseline levels for this task^69^.

Determining differences due to biological sex in other animal models of ALS/FTD is more challenging due to confounding factors such as genetic background and transgene expression levels. Nonetheless, some studies have reported sex-specific behavioural differences in mouse models of ALS and FTD. A transgenic TDP-43^A315T^ mutant mouse shows earlier disease onset and more rapid disease progression in males than females, accompanied by reduced lifespan^33^, although females perform worse in spatial learning tasks^34^. Female TDP-43^M323K^ knock-in mice harbouring a missense mutation in the endogenous *Tardbp* gene display an age-dependent decrease in grip strength, while males are normal^35^. Mouse models of other ALS and FTD-linked genes present a more complex picture. Males overexpressing human mutant superoxide dismutase 1 (SOD1^G93A^) show earlier disease onset than females^36^, although this is dependent on genetic background^37,38^. However, the opposite is observed in females harbouring an inducible D83G point mutation in murine *Sod1*, which demonstrate earlier impairment in Rotarod performance, though males reach end-stage sooner^39^. Female SOD1^G37R^ mice also have increased maladaptive axonal arborisation compared to males, which corresponds to neuronal loss and muscular denervation^40^. In contrast, transgenic female mice expressing the G118V mutation in profilin1 (*PFN1*) reach end-stage earlier than males, although age of disease onset is unaffected by sex^41^. Similarly, female transgenic mice expressing a mutant form of chromosome 9 open reading frame 72 (*C9ORF72*), the most common known genetic cause of ALS-FTD^3^, have reduced body weight while male mutants are unaffected^42^. This suggests an intricate regulation of sex-specific behaviours in diverse animal models.

Genetic studies in humans also suggest that biological sex influences the phenotype of both ALS and FTD. Mutations in *TARDBP*, while rare, appear to be more penetrant in male than female patients^21^. Conversely, mutations in *C9ORF72* have been shown to be more commonly seen in women than men with ALS^43^, although this difference is not observed in *C9ORF72*-related FTD^43^. Mutations in *progranulin* (*GRN*) are also more common in women^43^, and mutations in *T-cell restricted intracellular antigen 1* (*TIA1*) have, to date, been found exclusively in women^44^. Mutations in *microtubule-associated protein tau* (*MAPT*) by contrast are not specific to either sex^43^.

A likely explanation for sex differences in neurodegenerative disease is the role of reproductive hormones. Oestrogens, specifically 17β-oestradiol, can exert neuroprotective effects in both males and females by signalling through oestrogen receptors, which are widely distributed in the brain^45^. These effects range from maintenance of cognition and response to injury, to dendritic spine maturation and adult neurogenesis^46^. This is in keeping with findings from patients with ALS in whom the sex differences in incidence and prevalence diminish with age^4^. This may well be due to the reduction in levels of oestrogen in post-menopausal women^5^.

The protective effects of sex hormones are not only restricted to neurons, which constitutively produce oestradiol, but also glia^45^. Astrocytes show sex differences in their development, number and morphology, in addition to functional characteristics like glutamate uptake and their response to cannabinoids, gonadotrophic hormones, and harmful stimuli such as environmental toxins^47^. Microglia, the innate immune cells of the central nervous system, develop sex-specific transcriptional differences in adulthood, although it is debated which sex develops a more pro-inflammatory phenotype^48^. This may contribute to sex-specific differences in ALS-FTD, given that microglia play key roles during development and ageing, express high levels of the FTD-linked gene *GRN*^49^, and have been implicated in disease pathogenesis by several mouse models of ALS^50–52^.

The effects of sex hormones on brain mitochondrial metabolism have also been well documented^53^. For example, 17β-oestradiol can transcriptionally regulate and increase the function of components of the respiratory chain while reducing oxidative stress in brain mitochondria. Cerebral expression of the oestrogen receptors ERα and ERβ is also sexually dimorphic^53^. Incidentally, female transgenic mutant *SOD1* mice have delayed onset of mitochondrial dysfunction in the spinal cord compared to males^54^, possibly resulting from ERα-dependent activation of the mitochondrial unfolded protein response^55^. This transcriptional programme for restoration of proteostasis can also be activated by TDP-43^56^. Hormonal regulation may also affect disease course in ALS through regulation of the expression of a group of muscle-specific microRNAs^57^. The effects of progesterone and testosterone on the brain are, however, more elusive, with multiple mechanisms both conferring neuroprotection and enhancing neurodegeneration^58–63^.

Abundant evidence indicates sexual dimorphism in varied neurodegenerative diseases. Males have a two-fold increased risk of Parkinson’s disease, and also present with more marked non-motor symptoms compared to females^64^. In contrast, females are more likely to develop Alzheimer’s disease^65^, particularly those with the *APOEε4* allele^66^, and women with Huntington’s disease show a faster rate of progression than men^67^. Our findings add to a growing body of evidence suggesting that the influence of biological sex in neurodegenerative diseases is complex, resulting not only from genetic architecture, age, epigenomic and transcriptomic factors, but also from the effects of reproductive hormones on glial and neuronal cells, and organelle function. We conclude that the TDP-43^Q331K/Q331K^ knock-in mouse, which displays sex-specific behavioural differences, can be utilised as a tool to investigate female resistance to ALS-FTD and thereby help towards developing therapies for this disease spectrum.

## Methods

### Mouse model and genotyping

Mice were generated using CRISPR/Cas9 mutagenesis as described previously^23^ and maintained on a C57BL/6J background by crossing with wild-type animals. Animals were bred in a specific pathogen free environment and transferred to a conventional facility under a 12-h light/dark cycle. Cages (36 × 21 × 18.5 cm) were lined with fine sawdust (eco-pure flakes 6, Datesand, UK), a plastic house was placed in each cage and paper wool (Datesand, UK) was used as bedding material. All mice were singly housed due to the use of running wheels in the home cage, with food (Harlan, UK) and water available *at libitum*. Animals were genotyped as described previously^23^. All experiments were conducted in accordance with the United Kingdom Animals (Scientific Procedures) Act (1986) and the United Kingdom Animals (Scientific Procedures) Act (1986) Amendment Regulations 2012, and also reviewed and approved by the University of Sheffield Animal Welfare and Ethical Review Body (AWERB). Power calculations were determined as described previously^23^. All of the behavioural testing was carried out on the same cohort of female mice. One wild-type and one mutant mouse developed skin conditions at 1 year of age and were humanely culled on compassionate grounds. One wild-type and one mutant mouse lost 20% of their bodyweight nearing the 20-month time point and were culled on compassionate grounds. One additional mutant mouse was found to be unwell at 20 months of age and was culled.

### Body weight and food intake

Animals were weighed weekly in the morning as previously described^23^. Food intake was monitored at various timepoints by weighing the food in the hopper, then re-weighing approximately 72 h later. During the 72-h period, sawdust in the cage was replaced with paper towelling to ensure any small pieces of food dropped from the top of the hopper could be included for weighing.

### Marble burying

The marble burying assay was conducted as described previously^23^, except that different cages were used. Briefly, all testing was conducted in the morning and blind to genotype in cages of size 33 × 21 × 19 cm (Tecniplast) with fresh sawdust (Datesand, grade 6) placed to a height of ~ 8 cm. Ten glass marbles (1 cm) were placed evenly across the bedding. A single mouse was placed in each of the cages, the lids replaced, and left undisturbed for 30 min under white light. Mice were then removed, and the number of marbles buried by at least two thirds was scored.

### Catwalk gait analysis

The Catwalk gait analysis system 7.1 (Noldus Information Technology B.V., Netherlands, https://www.noldus.com/catwalk-xt) was used to capture gait parameters at 8, 10, 12, 14, and 20 months of age as previously described^38^. Mice were placed on the glass floor of the catwalk system in complete darkness and left to walk/run freely. Whenever possible, six continuous runs were recorded, with the three best runs being selected for analysis. Catwalk software 7.1 was used to label each paw print during each run and analyse the gait parameters of the mice.

### Running wheel analysis

The running wheel set-up was based on an in-house protocol described previously^68^. Each cage contained a 37.8 cm circumference Fast Trac running wheel (LBS Biotech, UK) mounted at 25˚ below horizontal, on a 4 cm fixed post. The wheel was placed in the corner of each cage where the circumference was 5–10 mm from the edge of two perpendicular sides of the cage. A 5 × 10 mm neodymium magnet was glued to the underside of each Fast Trac wheel and a bicycle computer (Cateye Velo, Japan) with reed switch was fixed to the side of the cage. Time spent running, distance run, and average running speed were recorded daily.

### Compound muscle action potential (CMAP) amplitude testing

Mice were placed under gaseous anaesthesia (1–2% isoflurane), with body temperature maintained using an electric heat pad (CWE, USA), and fur from the left hindlimb and lower back was removed. All recordings were made using a Dantec Keypoint Focus EMG System (Optima, UK) as previously described^38^. A grounding electrode was placed in the base of the tail (Ambu Neuroline, UK), and ring recording electrodes were placed circumferentially around the distal hindlimb muscles (Alpine Biomed, Denmark), layered with Ten20 nerve conductive paste (Pulse Medical Ltd, UK). CMAPs were acquired by applying a single, square wave electrical impulse of 0.1 ms duration to the sciatic notch using twisted pair subdermal electrodes (Ambu Neuroline, UK). The position of the subcutaneous stimulating electrodes was trialled to ensure that a response was obtained using a stimulating current of 1–2 mA. The stimulation intensity was then increased until no further increase in amplitude was seen and a supramaximal response elicited.

### Statistical analyses

All data was analysed using GraphPad Prism version 8 (https://www.graphpad.com/scientific-software/prism/). Statistical significance was determined by two-way analysis of variance (ANOVA) with or without repeated measures with Sidak correction for multiple testing, mixed effects analysis, or Student’s t-test. Results having *P* < 0.05 were considered significant in every analysis.

Please note that this methods section was adapted from the PhD thesis of the first author, which can be found at https://etheses.whiterose.ac.uk/id/eprint/16694 re-used here under a creative commons CC BY-NC-ND 2.0 UK license (https://creativecommons.org/licenses/by-nc-nd/2.0/uk/).
