# Author Correction: Female sex mitigates motor and behavioural phenotypes in TDP-43^Q331K^ knock-in mice

**DOI:** 10.1038/s41598-021-90064-2

**Published:** 2021-05-20

**Authors:** Jodie Watkins, Anshua Ghosh, Amy F. A. Keerie, James J. P. Alix, Richard J. Mead, Jemeen Sreedharan

**Affiliations:** 1 Department of Neuroscience, School of Medicine, Sheffield Institute for Translational Neuroscience, University of Sheffield, Sheffield, S10 2HQ UK; 2 Maurice Wohl Clinical Neuroscience Institute, Institute of Psychiatry, Psychology and Neuroscience, King’s College London, 5 Cutcombe Road, London, SE5 9RX UK

Correction to: *Scientific Reports* 10.1038/s41598-020-76070-w, published online 05 November 2020

The Acknowledgements section in this Article is incomplete.

“We thank staff of the Babraham Institute Experimental Unit and Biological Services Unit at the University of Sheffield for technical assistance. J.S. gratefully acknowledges support from the Motor Neuron Disease Association, the Medical Research Council UK, the Lady Edith Wolfson Fellowship Fund, the van Geest Foundation, the Rosetrees Trust, Alzheimer’s Research UK, and the Psychiatry Research Trust. This work was partly funded by Motor Neuron Disease Association grants awarded to R.M. (SITraN/Apr13/983-797 and SITraN/Jul16/987-797) and to R.M. and J.W. (Mead/Jun16/900-790). J.J.P.A. is funded by a National Institute for Health Research Clinical Lectureship. J.W. was funded by a University of Sheffield PhD scholarship.”

should read:

“We thank staff of the Babraham Institute Experimental Unit and Biological Services Unit at the University of Sheffield for technical assistance. J.S. gratefully acknowledges support from the Motor Neuron Disease Association, the Medical Research Council UK (MR/K010611/1), the Lady Edith Wolfson Fellowship Fund, the van Geest Foundation, the Rosetrees Trust, Alzheimer’s Research UK, and the Psychiatry Research Trust. This work was partly funded by Motor Neuron Disease Association grants awarded to R.M. (SITraN/Apr13/983-797 and SITraN/Jul16/987-797) and to R.M. and J.W. (Mead/Jun16/900-790). J.J.P.A. is funded by a National Institute for Health Research Clinical Lectureship. J.W. was funded by a University of Sheffield PhD scholarship.”

